# Echo-Planar J-resolved Spectroscopic Imaging using Dual Read-outs: Implementation and Quantitation of Human Brain Metabolites

**DOI:** 10.1038/s41598-017-03121-0

**Published:** 2017-06-08

**Authors:** Manoj K. Sarma, Rajakumar Nagarajan, Zohaib Iqbal, Paul M. Macey, M. Albert Thomas

**Affiliations:** 1 Department of Radiological Sciences, David Geffen School of Medicine at UCLA, Los Angeles, California USA; 2 UCLA School of Nursing, Los Angeles, California USA; 3 Brain Research Institute, David Geffen School of Medicine at UCLA, Los Angeles, California USA

## Abstract

Attempts have been made to reduce the total scan time in multi-dimensional *J*-resolved spectroscopic imaging (JRESI) using an echo-planar (EP) readout gradient, but acquisition duration remains a limitation for routine clinical use in the brain. We present here a significant acceleration achieved with a 4D EP-JRESI sequence that collects dual phase encoded lines within a single repetition time (TR) using two bipolar read-out trains. The performance and reliability of this novel 4D sequence, called Multi-Echo based Echo-Planar *J*-resolved Spectroscopic Imaging (ME-EP-JRESI), was evaluated in 10 healthy controls and a brain phantom using a 3 T MRI/MRS scanner. The prior knowledge fitting (ProFit) algorithm, with a new simulated basis set consisting of macromolecules and lipids apart from metabolites of interest, was used for quantitation. Both phantom and *in*-*vivo* data demonstrated that localization and spatial/spectral profiles of metabolites from the ME-EP-JRESI sequence were in good agreement with that of the EP-JRESI sequence. Both in the occipital and temporal lobe, metabolites with higher physiological concentrations including Glx (Glu+Gln), tNAA (NAA+NAAG), mI all had coefficient of variations between 9–25%. In summary, we have implemented, validated and tested the ME-EP-JRESI sequence, demonstrating that multi-echo acquisition can successfully reduce the total scan duration for EP-JRESI sequences.

## Introduction

Magnetic Resonance Spectroscopic Imaging (MRSI)^1, 2^ is a technique that combines features of both imaging and spectroscopy, and is increasingly being used to study human brain metabolism^3–7^. MRSI involves recording spectra from multiple brain regions either from a single slice or a volume containing many slices^8^ with increased spatial coverage. Similar to MRI, phase encoding gradients are used by the conventional MRSI sequence in order to encode spatial distribution of tissue metabolite concentrations. To allow the spectral acquisition from a volume covering the whole brain, a partial or maximum echo or a free induction decay (FID) is recorded per phase encoding step without the presence of any gradient. Due to the incremental phase encoding steps, total duration can be an hour or longer depending on the desired spatial resolution. The total measurement time required to sample a spatially-encoded three dimensional (3D) volume is calculated as, *N*_*x*_ × *N*_*y*_ × *N*_*z*_ × TR × *N*_*av*_, where *N*_*x*_, *N*_*y*_, *N*_*z*_ are the phase encoding steps along the three spatial dimensions (x, y, z), TR is the repetition time, and *N*_*av*_ is the number of averages. A simple two dimensional (2D) MRSI acquisition with average = 1, pixel resolution = 32 × 32, and TR = 1 s will require a scan time of over 17 min. To achieve an adequate signal-to-noise ratio (SNR), multiple averages may be required, which will push the total acquisition duration to impractical levels. Clinically feasible sequences therefore require novel MRSI techniques that reduce the scan time.

In addition to the long acquisition time, these one-dimensional (1D) spectral based MRSI sequences suffer from overlapping peaks. Taking advantage of the J-coupling interactions between protons of metabolites and a better separation of the resonance lines by adding an extra spectral dimension as accomplished in localized correlated spectroscopy (L-COSY)^9^ and *J*-resolved spectroscopy (JPRESS)^10, 11^, spectral overcrowding can be reduced which helps in accurate quantification of several low concentration metabolites. Brain *in*-*vivo* studies involving single voxel (SV) based JPRESS show spectral separation in many clinically-relevant metabolites often difficult to resolve with 1D MRS, such as lactate (Lac), glutathione (GSH), glutamate/glutamine (Glx) and myo-inositol (mI)^11–14^. Integration of the multi-voxel encoding techniques with the multidimensional sequence has the potential to provide spatial information about a range of metabolites obscured in 1D MRS^1, 15^. One of the major hurdles in clinical implementation of these multi-dimensional MRSI sequences is also longer scan time.

To overcome these scan time limitations, variants of the basic MRSI sequence have been implemented. Echo-planar readout gradient, first proposed by Mansfield^16, 17^ and subsequently implemented by Posse *et al*. using proton echo-planar spectroscopic imaging (PEPSI)^18, 19^ also known as echo-planar spectroscopic imaging (EPSI)^20^, has greatly shortened the acquisition of 2D/3D MRSI. In EPSI, a time varying readout-gradient train encodes one spatial and one spectral (temporal) dimension during a single readout, leaving the remaining spatial dimensions to be incrementally phase encoded sequentially resulting in an acceleration of N_x_ or N_y_ times. Such an acceleration in scan time makes it feasible to acquire spatially resolved multi-dimensional spectral data in a clinical setting^21^, increase spatial resolution, or collect 3D data sets^20^. Four-dimensional (4D) echo-planar J-resolved spectroscopic imaging (EP-JRESI)^22, 23^ is one such sequence which combines the increased spectral dispersion offered by 2D JPRESS with the speed advantage of EPSI readout and is capable of collecting 2D spectra with improved spectral resolution from multiple regions. The EPSI readout simultaneously acquires one spatial (*k*_x_) and one temporal (*t*_2_) dimension, leaving the rest (*k*_y_ and *t*_1_, respectively) to be recorded incrementally resulting in a scan time on the order of 20+ minutes.

Multi echo (ME) encoding schemes, like turbo spin echo (TSE) and fast spin echo (FSE)^24–26^, dramatically reduce the total scan duration in magnetic resonance imaging (MRI). Similar ME-based techniques hold the promise of greatly reduced scan times in MRSI and their applicability to spectroscopy has been demonstrated in 1D MRSI and Correlated Spectroscopic Imaging studies^27–30^. One of the problems with ME-based spectroscopy is the T_2_ decay diminishing signal with each echo, which may be a limiting factor in living tissues where T_2_ relaxation times are shorter^31, 32^. This limitation can be minimized by keeping the time between the different echoes as short as possible, but this in turn will limit the number of acquired spectral points and hence overall spectral resolution.

In this work, the major goals were as follows: 1) To implement a novel ME-EP-JRESI sequence for the study of human brain at 3 T, and to quantify the cerebral metabolites using the ProFit algorithm^33–36^; and 2) To evaluate ME-EP-JRESI in healthy controls and a phantom. Phantom scans are presented here from a gray matter brain phantom to study the acceleration of scan time compared with EP-JRESI and to show that quantitation results using the ProFit algorithm are not affected by the multi echo readout. *In*-*vivo* scans from healthy controls are presented to show that spatial and spectral quality is maintained despite the loss of spectral resolution.

## Materials and Methods

**Figure 1:**
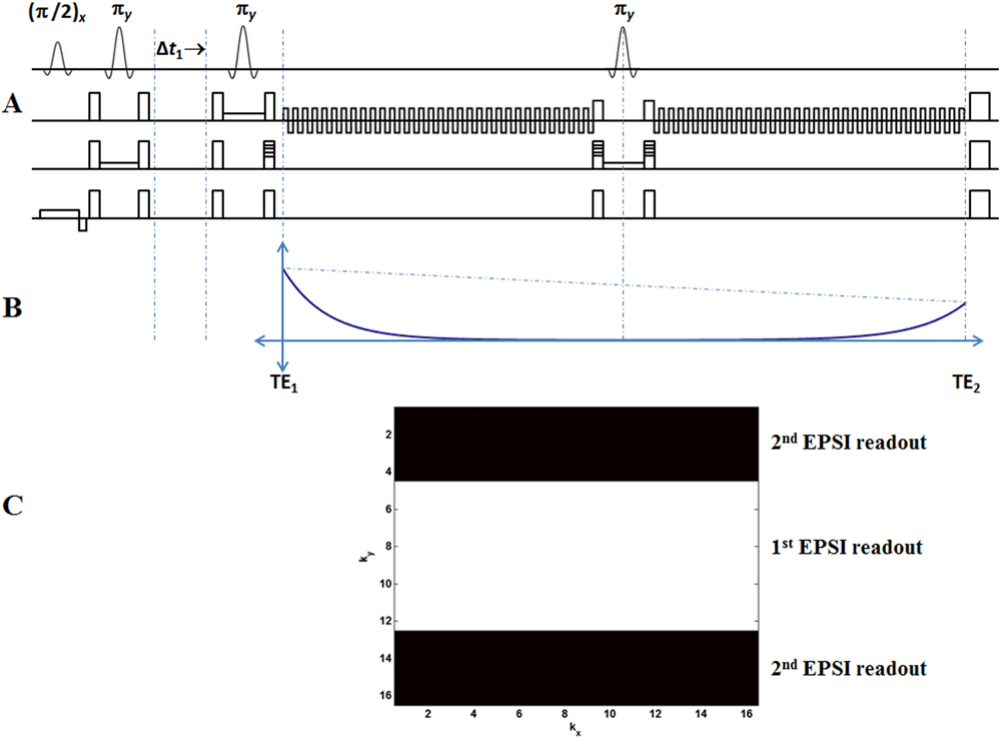
(**A**) A schematic diagram of the PRESS-based ME-EP-JRESI sequence, showing the three localization pulses and two echo-planar readouts separated by a slice selective 180° pulse; (**B**) shows the effect of the spin echo on the overall shape of the signal envelope, where the solid line represents the T_2_^*^ decay and the dashed line represents the decay due to T_2_; (**C**) shows the *k*-space acquisition by the two EPSI read-outs.

Using the Siemens VB17a compiler, a pilot version of the 4D ME-EP-JRESI sequence modified from the ME-EPSI sequence^29^ was implemented on a 3 T Trio-Tim MRS/MRI scanner (Siemens Medical Solutions, Erlangen, Germany). An illustration of the multi-voxel 4D ME-EP-JRESI sequence can be seen in Fig. 1. At the heart of the ME-EP-JRESI sequence is the JPRESS module containing three slice-selective radio frequency (RF) pulses (90°–180°-Δt_1_-180°) with a time delay before the last 180° RF pulse^11, 12^ to collect the indirect spectral dimension t_1_. The time delay was incremented by (Δ*t*_1_) continuously increasing the effective echo time (TE) of repetitions over the t_1_ increments. Two bipolar EPSI read-out trains differently phase-encoded along the spatial dimension and separated by a slice selective refocusing 180° pulse were included in the ME-EP-JRESI technique to accelerate the acquisition by collecting two *k*-space lines per TR. The first EPSI read-out sampled the magnetization after the last localization pulse starting at echo time *TE*_1_. The decaying magnetization during the first read-out train was then refocused using the slice-selective 180° pulse and another line was phase encoded in k-space which was measured by the second EPSI read-out. Subsequently, two phase-encoding steps along the same spatial domain were accomplished within the same TR and the whole cycle was repeated again to acquire another pair of *k*-space line. The central areas of *k*-space, where more signal is expected, were acquired by the first EPSI readout and outer areas were acquired by the second readout in each TR as described by Duyn *et al*.^27^ previously. The dephasing and refocusing of magnetization during two encoding steps are shown in Fig. 1(B) with Fig. 1(C) showing the *k*-space acquisition. A more detailed discussion on implementation of multi-echo and its effect on the point-spread function (PSF) can be found elsewhere^29, 30^. All data were acquired using a twelve-channel ‘receive’ head coil and RF pulses were transmitted using a quadratic body coil.

### Phantom

A one liter brain phantom was prepared containing the following metabolites (pH = 7.3) at physiological concentrations as reported in healthy human brain^37^: creatine (Cr, 7 mM), N-acetylaspartate (NAA, 8.9 mM), N-acetylaspartylglutamate (NAAG, 0.51 mM), glutamate (Glu, 12.5 mM), glutamine (Gln, 2.5 mM), mI (4.4 mM), free choline (Cho, 0.9 mM), phosphorylcholine (PCh, 0.6 mM), taurine (Tau, 1.8 mM), GSH (2.0 mM), glucose (Glc, 1.0 mM), Lac (1.0 mM), γ-aminobutyrate (GABA, 0.7 mM), aspartate (Asp, 2.1 mM), phosphoethanolamine (PE, 1.0 mM), threonine (Thr, 0.3 mM), alanine (Ala, 1 mM), valine (Val, 1.0 mM), isoleucine (Ile, 1.0 mM), leucine (Leu, 1.0 mM), and lysine (Lys, 1.0 mM). The amino acids: Ala, Thr, Val, Leu, Ile, Lys were included in the brain phantom to represent the macromolecule (MM) baseline signal. Multi slice T1-weighted MRIs were acquired first for localization followed by ME-EP-JRESI. The ME-EP-JRESI phantom scans were recorded using the following parameters: field-of-view (FOV) = 16 × 16 cm^2^, matrix size = 16 × 16, a slice thickness of 2 cm, 100 t_1_ increments with Δ*t*_1_ = 1 ms, 256 bipolar echo-pair with a repeat time of 0.84 ms resulting in a *TE*_2_ = 472.5 ms for a difference in echo time between the first and second readout of Δ*TE*_21_ = 442.5 ms. A maximum echo sampling scheme was applied and after post-processing for each acquisition effective Δ*t*_1_ was 2 ms and reconstructed bandwidths in the direct (F_2_) and indirect (F_1_) spectral dimensions were 1190 and ±250 Hz, respectively. With TR/TE = 2 s/30 ms and 1 average, the total duration for acquiring a 4D ME-EP-JRESI phantom scan was approximately 1 hour 10 minutes. B_0_ homogeneity of the PRESS-selected VOI was optimized with a combination of standard automatic global shim provided by the manufacturer (Siemens) and manual adjustment of the three linear gradients to achieve a full width of the water resonance at half maximum (FWHM) of 9 Hz or less (in magnitude mode). During the shimming process, the x, y, and z gradients were adjusted by minimizing the FWHM and maximizing the peak intensity while simultaneously preserving the peak shape. It is to be noted that a small improvement in the shim can make a huge difference in the quality of the spectra. Following frequency adjustment, water-selective suppression was optimized by using the WET (water suppression enhanced through T_1_) technique^38^ which uses three water-suppression pulses. A non-water suppressed scan was also acquired to correct for eddy-currents^39^ generated by the EPSI readout and also as a reference for coil combination. Non-water-suppressed ME-EP-JRESI data was recorded only along k_x_, k_y_ and detected spectral (t_2_) dimensions. In total, we acquired 10 *in vitro* measurements. To compare the quality of the ME-EP-JRESI data, EP-JRESI measurements were also collected using the same parameters as of ME-EP-JRESI scans. The total scan duration for water suppressed EP-JRESI was approximately 2 hours 20 min.

### *In*-*Vivo*

To assess clinical applicability, the 4D ME-EP-JRESI sequence was tested in the brain of 10 healthy controls (age of 50.9 ± 9.6 years). Written informed consent was obtained prior to study participation, and all data were acquired in accordance with the UCLA Institutional Review Board (IRB) approved protocol. Before evaluating the ME-EP-JRESI sequence, 3D high resolution T_1_-weighted images for localization were collected using a Magnetization Prepared Rapid Gradient Echo (MPRAGE) pulse sequence. 4D ME-EP-JRESI was performed over an axial slice covering frontal, basal ganglia and occipital regions using the same parameters as the phantom scans with the exception that the TR was decreased to 1.5 s and 64 *t*_1_ increments was used for a scan time of around 13 minutes. Outer volume saturation bands were included outside the PRESS volume of interest. Similarly, to the *in vitro* scans the voxel shim and water suppression were performed manually, and a line width of ~20 Hz was achieved. This was followed by a non water-suppressed scan with only the first *t*_1_ increment, adding 18 s to the total scan duration.

### Post-processing and Quantitation

**Figure 2:**
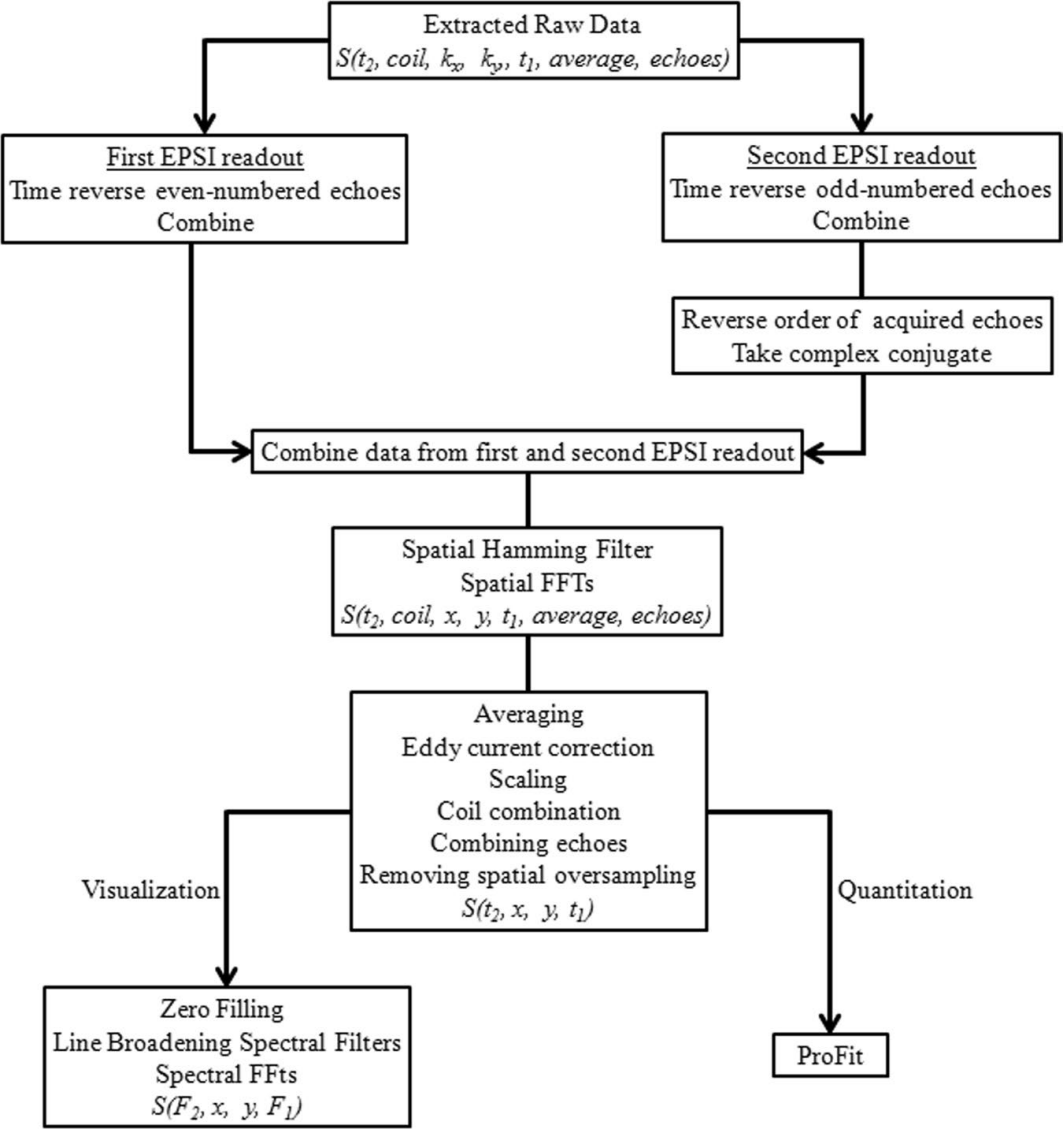
Flowchart depicting different processing steps for ME-EP-JRESI data.

All data were extracted and post-processed using a custom MATLAB-based library of programs (The Mathworks, Natick, MA, USA). The presence of two EPSI readout trains in ME-EP-JRESI required special considerations in post-processing. The bipolar nature of the EPSI readout gradients generates two different sets of *k*-space trajectories that produce mirror-images after applying a Fast Fourier Transform (FFT) (in real space). This is true for the EP-JRESI sequence also. This can be fixed by time-reversing the even-numbered (second echo) gradient echoes and combining them together. For the second EPSI readout in ME-EP-JRESI after the spin echo, the first gradient echo corresponds to the same trajectory in *k*-space as the last (even-numbered) gradient echo in the first EPSI readout. This requires that all odd-numbered echoes in the second EPSI readout need to be time reversed. As shown in Fig. 1(B), the maximum amplitude for the second EPSI readout in the ME-EP-JRESI sequence occurs at the end where as in the first EPSI readout it arises in the beginning. As a result, the order of the second EPSI readout needs to be reversed so that all echo maxima line up together in k-space. But this order reversal may cause mirroring, and therefore the complex conjugate needs to be taken of the second EPSI readout after the reversal of the order. A flowchart of the post-processing steps of the ME-EP-JRESI data is shown in Fig. 2. After sorting out all the data, a spatial Hamming filter was applied to the data in *k*-space denoted as *S*(*t*_2_, *coil*, *k*_*x*_, *k*_*y*_, *t*_1_, *average*, *echoes*) and FFTs were applied along the *k*_*x*_ and *k*_*y*_ dimensions to yield spatial dimensions x and y with the spectral dimensions still in time-domain, *S*(*t*_2_, *coil*, *x*, *y*, *t*_1_, *average*, *echoes*). This was followed by post-processing through a series of steps including scaling by a constant value, combining echoes, removing spatial oversampling along the read-out direction (*k*_*x*_), coil combination, and eddy current correction^32^. The final dataset comprised of a 4D matrix arranged in the format *S*(*t*_2_, *x*, *y*, *t*_1_). EP-JRESI phantom data were also post-processed using the same steps other than the multi-echo aspects. For visualization, zero-filling by a factor of two was applied along both spectral dimensions followed by skewed sine-bell filters (skew = 0.5) for line-broadening and 2D FFT along the two time dimensions to yield the spectral dimensions F_1_ and F_2_, respectively. The multi-voxel plotting was done using custom MATLAB code and the extracted 2D J-resolved spectra were plotted using the NMR-specific plotting program, Felix-2000 (Felix NMR Inc., San Diego, CA).

*In*-*vivo* and *in*-*vitro* metabolite concentrations were estimated using the Profit algorithm^33^, optimized for processing the Siemens data, based on a linear combination of 2D model spectra. ProFit uses a non-linear outer loop and an inner linear least-squares fit for the concentrations and incorporates basis files representative of each individual metabolite. Prior to fitting the ME-EP-JRESI data, zeroth-order phase correction and frequency shifts were applied to the extracted 2D J-resolved spectra. A prior knowledge basis set was simulated with the GAMMA (general approach to magnetic resonance mathematical analysis) library^40^, using chemical shifts and J-coupling values reported in the literature^37^, and was post-processed in a similar fashion as the recorded data^14^. Separate basis sets were created each for *in*-*vivo* and phantom data. While the phantom basis set contained the same metabolites as the original phantom, the *in*-*vivo* basis set contained 19 metabolites: Cr, NAA, PCh, Cho, glycerylphosphocholine (GPC), Asp, GABA, Glc, Gln, Glu, GSH, Lac, mI, NAAG, PE, scyllo-inositol (sI), ascorbate (Asc), glycine (Gly), and Tau. MM signals are prominent in short TE based MRSI and are challenging to quantify *in vivo*. For a more reliable quantification of the *in*-*vivo* metabolites, we therefore included the following amino acids (representing MM) /lipids in the basis set: Ala, Thr, Val, Leu, Ile, Lys, methylene singlet (palmitic acid) at 1.32 and 1.26 ppm, palmitic acid methyl at 0.89 and 1.26 ppm, fat at 1.64 and 2.36 ppm.

Metabolite concentrations were reported as ratios with respect to the Cr peak at 3.0 ppm (*S*/*S*_*Cr*_). No correction for T_1_ and T_2_ decay was used. For each metabolite, the quality of the fitting was individually evaluated using the Cramer-Rao Lower Bound (CRLB) values^41^. Criteria for rejection of spectra were based on patient movement, or poor water suppression (FWHM > 30 Hz). Any metabolite values with CRLB >20% in the ProFit quantitation were not considered in the analysis (both *in*-*vivo* and phantom). Furthermore, all spectra with a Cr 3.91 ppm to Cr 3.03 ppm ratio of >1.3 were discarded from the ProFit results. Ideally the ratio of the two Cr peaks, which were included as separate basis set metabolites, is unity^33^. A high value reflects poor spectra caused by bad water suppression. Metabolite maps for NAA, Glx, Cr and Cho were obtained by integrating the spectra over the peak ranges: NAA (F_2_ = 1.8–2.2 ppm, F_1_ = ±15 Hz), Glx (F_2_ = 2.3–2.5 ppm, F_1_ = ±15 Hz), Cr (F_2_ = 2.9–3.1 ppm, F_1_ = ±15 Hz) and Cho (F_2_ = 3.1–3.3 ppm, F_1_ = ±15 Hz).

### Statistical analysis

All results are expressed as means ± standard deviation (SD) and coefficient of variation (CV). The agreement between ME-EP-JRESI and EP-JRESI measurements for phantom data was assessed with the Bland-Altman method^42^, in which the differences between metabolite ratios for the two measurements were plotted against their means with repeatability coefficient (RC), which allows calculation of the 95% limits of agreement. The repeatability coefficient was defined as 1.96 times the standard deviation of the mean difference between two measurements. Reproducibility and reliability were assessed by intraclass correlation coefficient (ICC), based on 2-way random-effects analysis of variance. A correlation analysis was also done using selected ratios of the major metabolites from the ProFit quantified EP-JRESI and ME-EP-JRESI data for a boundary voxel. Statistical analysis was performed with SPSS software (version 22.0, SPSS Inc., Chicago, IL).

## Results

### Phantom

**Figure 3:**
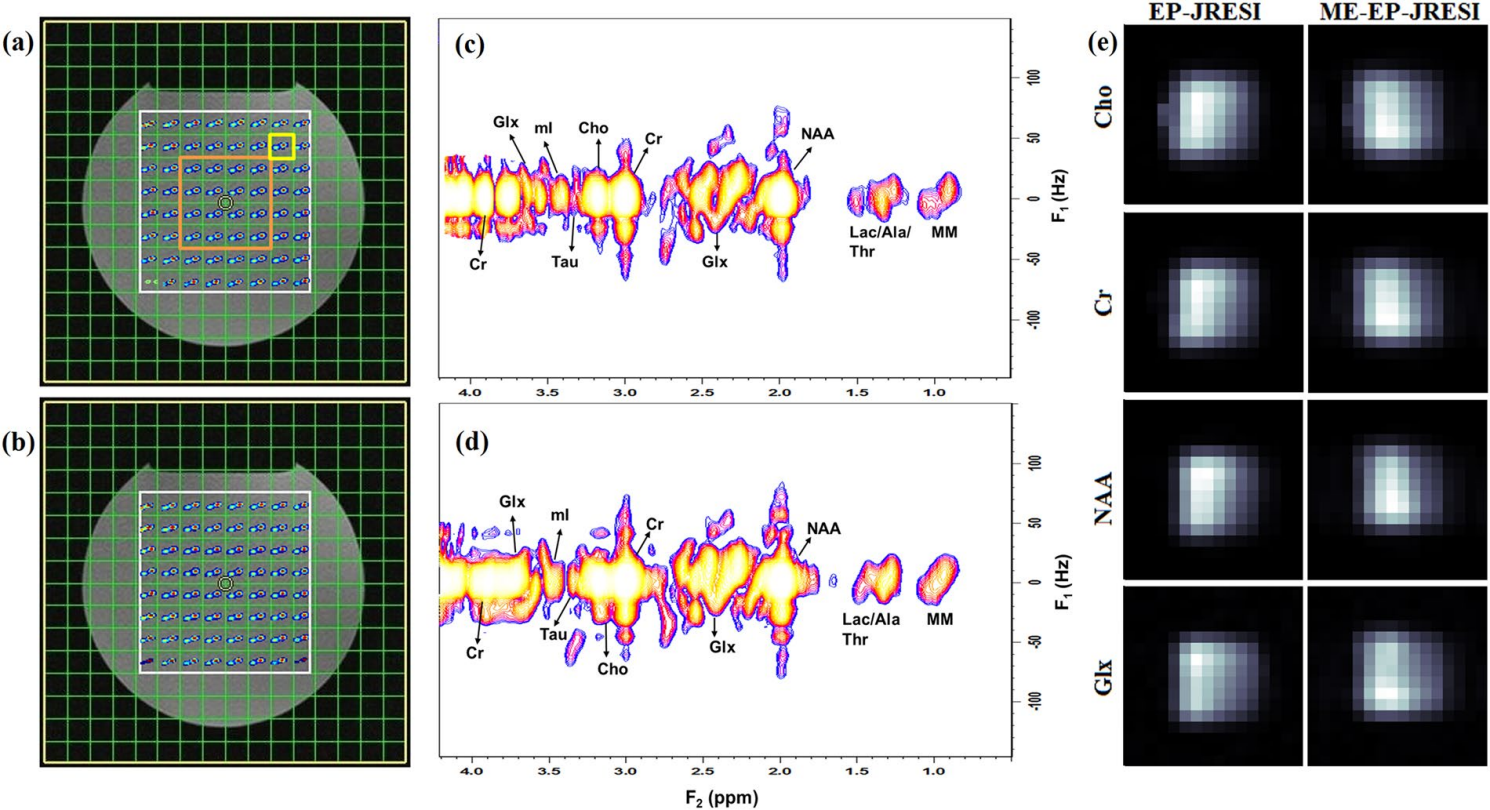
The multi-voxel spatial distribution of 2D peaks of Cr/Cho (F_2_ = 2.9–3.3 ppm, F_1_ = ±15 Hz) overlaid on top of the *T*_1_-weighted axial MRI from (**a**) EP-JRESI scan, (**b**) ME-EP-JRESI scan; selected 2D *J*-resolved spectra (**c**) from EP-JRESI, (**d**) ME-EP-JRESI, extracted from the voxel in yellow box in (**a**); and (**e**) comparison of metabolite maps as measured by peak area integration obtained by summing the peak area over the range of the metabolites. The orange box in (**a**) shows the 4 × 4 voxel considered for Table 1. The white box in (**a**,**b**) indicates the PRESS localization for respective data.

Results from the gray matter phantom scan for the EP-JRESI and ME-EP-JRESI sequences are shown in Fig. 3. Figure 3(a,b) show the spatial distribution of the 2D peaks of Cr and Cho (F_2_ = 2.9–3.3 ppm, F_1_ = ±15 Hz) from EP-JRESI and ME-EP-JRESI overlaid on the T_1_-weighted axial MRI respectively. Figure 3(c,d) show extracted 2D spectra from one EP-JRESI and one ME-EP-JRESI measurement respectively. The metabolite map of the 2D peaks of singlets from Cr, Cho, NAA and J-coupled metabolite Glx from ME-EP-JRESI and EP-JRESI are shown in Fig. 3(e). The peaks were localized within the PRESS excitation volume with minimal leakage.

The reliability of the ME-EP-JRESI data was investigated using the ProFit algorithm. Table 1 shows a comparison of metabolite ratios with respect to Cr based on the ProFit quantitation of the ME-EP-JRESI and EP-JRESI data. The central 4 × 4 voxels of the excited volume for each sequence, marked by an orange box in Fig. 3(a), were fitted with ProFit. Concentrations were expressed as ratios with respect to Cr and averaged across the volume. Mean ratios and CV among the voxels were computed and only those metabolites fit with CRLBs <20% were included in the calculations. The ME-EP-JRESI spectra were of high quality, with metabolite ratios matching the EP-JRESI data, and were in good agreement with known values of the phantom metabolites. The mean ratios for most of the metabolites for EP-JRESI, and ME-EP-JRESI were within 20% of each other, which indicates the estimate precision across the volume of interest is not greatly affected by mixing spin echoes with multiple TEs. The inter-scan CV was small for the predominant singlets (<25%). Most other metabolites were detectable with CV values in the range of 20–50%. The CVs were slightly higher because of the larger number of voxels around the center (4 × 4) from each of the measurement were included in the calculation and also for the fact that some of the metabolites like PE, Gln, Ala, Glc are not reliably estimated by the ProFit algorithm. The number of voxels successfully fitted with a CRLB <20% for each metabolite in ME-EP-JRESI was almost the same with EP-JRESI.

**Table 1:** Summary of the ProFit quantified results for selected metabolites for the two sequences.

Metabolites	Expected Ratios (*S*/*S*_*Cr*_)	Experimental Ratios (*S*/*S*_*Cr*_)					
		ME-EP-JRESI			EP-JRESI		
		Mean	CV (%)	*N*_*inc*_	Mean	CV (%)	*N*_*inc*_
NAA	1.27	1.21	11.67	160	1.25	12.78	160
PCh	0.09	0.12	44.13	156	0.15	26.24	154
Cho	0.13	0.13	25.49	153	0.15	21.09	154
Asp	0.30	0.35	25.94	158	0.23	24.25	141
GABA	0.10	0.28	37.13	155	0.26	42.44	154
Gln	0.36	0.27	42.18	155	0.35	27.63	156
Glu	1.79	1.26	11.58	160	1.01	14.43	160
GSH	0.29	0.51	16.72	158	0.48	14.56	156
Lac	0.14	0.14	70.46	45	0.04	70.54	42
mI	0.63	0.87	23.90	160	0.60	13.96	160
NAAG	0.07	0.15	78.15	154	0.13	69.18	138
PE	0.14	0.14	67.92	97	0.10	75.87	60
Tau	0.26	0.25	39.02	139	0.24	32.23	156
Val	0.14	0.19	58.84	111	0.15	54.56	93
Lys	0.14	0.21	59.87	157	0.25	55.27	154
tNAA	1.34	1.35	8.06	160	1.36	10.56	160
tCho	0.21	0.25	13.96	160	0.29	16.41	160
Glx	2.14	1.52	9.77	160	1.36	13.36	160

The central 4 × 4 voxels of the excited volume, as shown in Fig. 3 with the orange box, were processed. Ratios are shown with respect to Cr. *N*_*inc*_ is the number of voxels in which ProFit reported CRLBs <20%. Only those voxels were included for the mean ratios and CVs.

**tCho = *Cho* + *GPC* + *PCh*, *tNAA* = *NAA* + *NAAG*, *Glx* = *Glu* + *Gln*.

**Figure 4:**
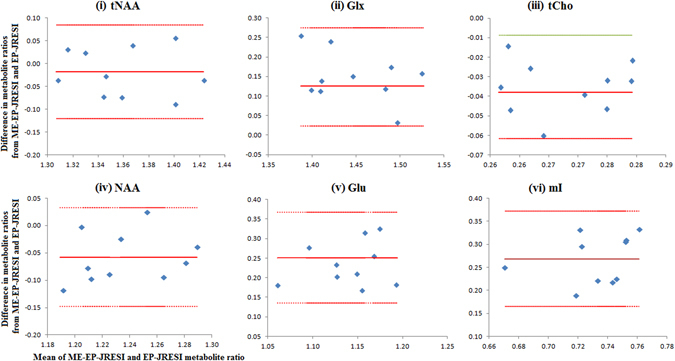
Bland-Altman analysis showing the agreement of phantom metabolite ratios (by ProFit) between the ME-EP-JRESI and EP-JRESI measurements. The red solid lines represent the average difference and the two dashed lines represent lower and upper limits of agreement. All the plots (i–vi) have the same x-axis (Mean of ME-EP-JRESI and EP-JRESI metabolite ratios).

Both ME-EP-JRESI and EP-JRESI showed good agreement using Bland Altman analysis (Fig. 4). The mean difference between the techniques for the major metabolites was between 1.3–20% with a RC ±3–5%. The mI ratios obtained from ME-EP-JRESI were relatively higher when compared EP-JRESI, with a mean difference of 30%. The ICC among the 10 phantom measurements for EP-JRESI and ME-EP-JRESI was 0.99 and 0.97 respectively, indicating that these measurements were highly reproducible.

**Figure 5:**
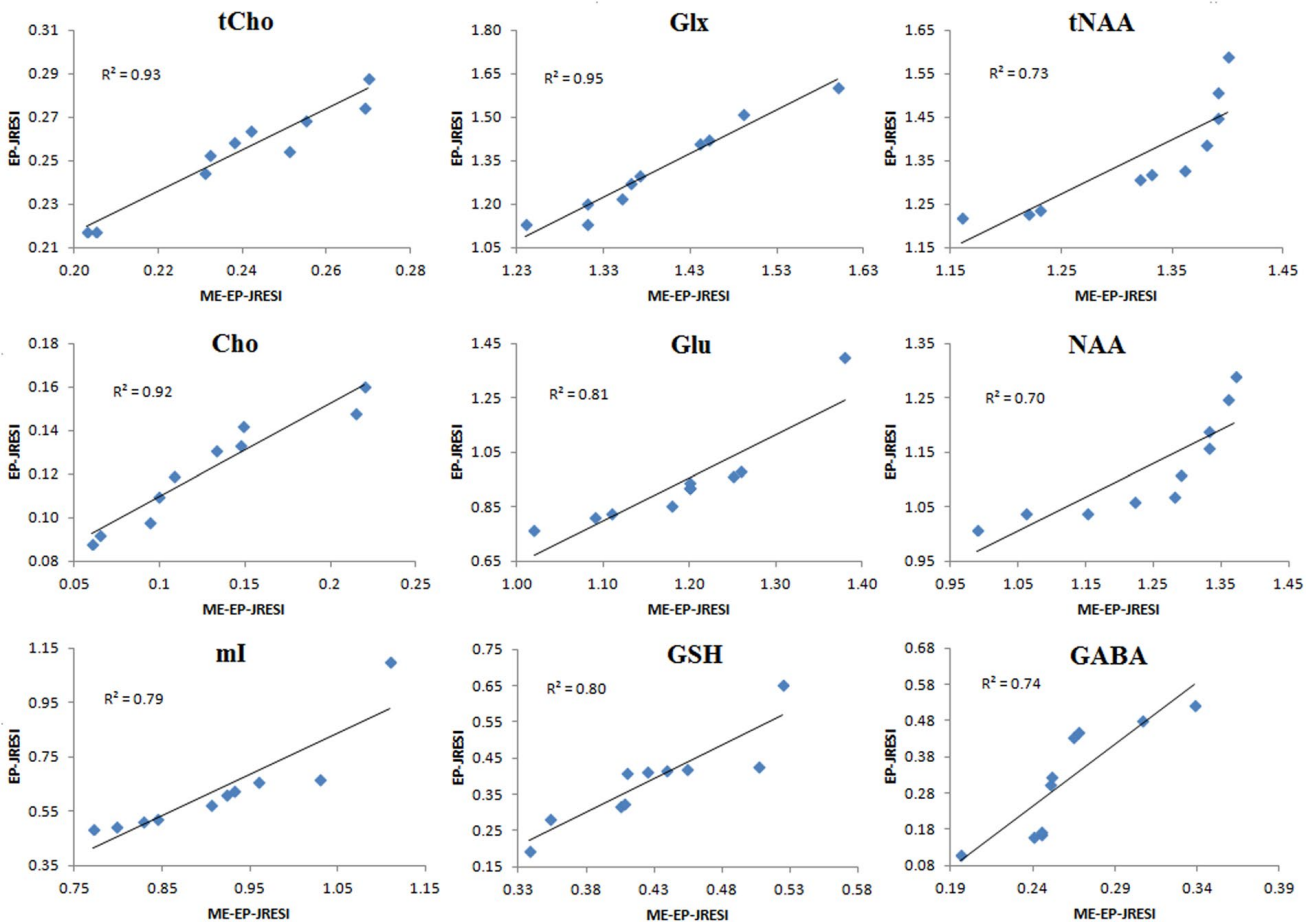
Correlations and R^2^ values between metabolite ratios of the phantom data from ME-EP-JRESI and EP-JRESI sequences for the 10 measurements.

Figure 5 shows the scatter plots of tCho, Glx, tNAA, Cho, Glu, NAA, mI, GSH, and GABA with linear fitting line from a boundary voxel marked by yellow box in Fig. 3(a) for the 10 measurements. Positive correlations were observed in all the major as well as low concentration metabolites, with *R* ^*2*^ values ranging from 0.7 to 0.95. Even for metabolites with shorter T_2_ constants like Glx, we found very high correlations (*R* ^*2*^ = 0.95 for Glx).

### Healthy Brain *In*-*vivo*

**Figure 6:**
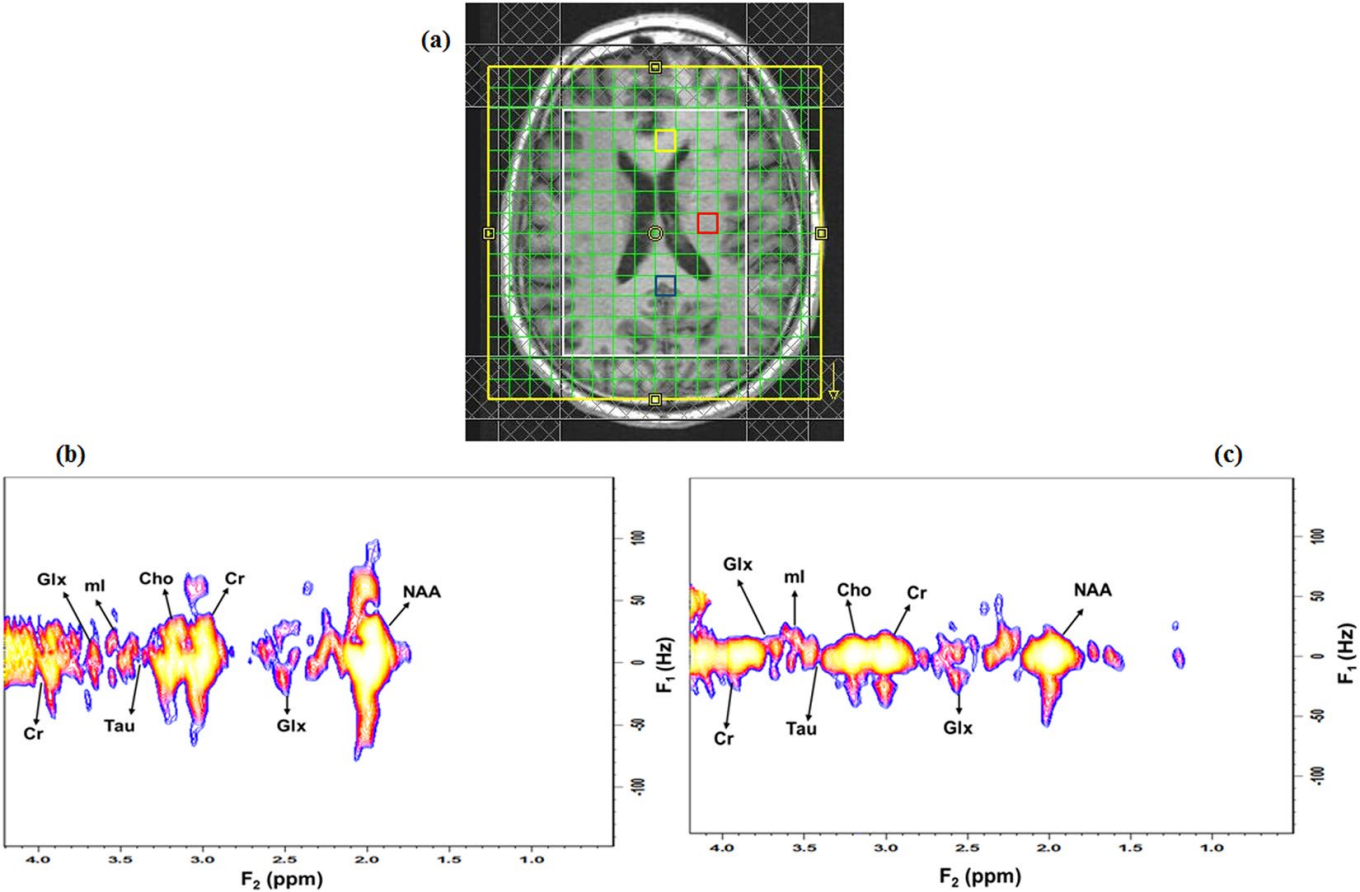
(**a**) *T*_1_-weighted axial MRI of a 59-year-old healthy brain with the white box indicating the PRESS localization, (**b**) selected 2D *J*-resolved spectra extracted from the mid occipital (voxel in blue) and, (**c**) mid frontal (voxel in yellow).

Figure 6(a) shows the T_1_-weighted MRI of a 59-year-old healthy brain with the voxel location for the 4D ME-EP-JRESI. The extracted 2D *J*-resolved spectra from the mid-frontal and mid-occipital, marked by the yellow and blue box respectively in the T_1_-weighted localizing image, are shown in Fig. 6(b,c). Table 2 shows the cerebral metabolite ratios with respect to Cr over the left temporal and mid-occipital regions (marked by the red and blue box respectively in Fig. 6(a)) in the healthy controls. Voxels in the mid-occipital region contained mainly grey matter, although some contribution from white matter is unavoidable. Left temporal voxels predominantly consisted of white matter. Most of the metabolites of interest were detected with good reproducibility as evidenced by the SD. Metabolites at higher physiological concentrations including Glx, tNAA, mI, and NAA showed 2D J cross-peaks with CVs between 13–25%. Some of the low concentration metabolites like Asp, GABA, Gln, PE, Tau also showed acceptable CVs < 30%. Ala and Glc had the poorest fit and were omitted from the Table 2.

**Table 2:** Summary of the *in*-*vivo* ProFit-quantified results for selected metabolites showing mean metabolite ratios (±SD) with respect to Cr in the mid occipital (voxel in blue in Fig. 6(a)) and left temporal lobe (voxel in red in Fig. 6(a)) of 10 healthy controls.

Metabolites	Mid Occipital (Mean ± SD)	Left Temporal (Mean ± SD)
NAA	1.77 ± 0.31	1.47 ± 0.24
Glu	1.55 ± 0.27	1.65 ± 0.43
mI	0.67 ± 0.16	1.03 ± 0.17
tNAA	2.52 ± 0.40	2.20 ± 0.43
Glx	1.89 ± 0.26	1.92 ± 0.33
NAAG	0.75 ± 0.38	0.79 ± 0.24
Asp	0.47 ± 0.13	0.80 ± 0.19
PCh	0.04 ± 0.02	0.07 ± 0.01
GPC	0.12 ± 0.04	0.11 ± 0.04
Cho	0.21 ± 0.05	0.25 ± 0.05
GABA	0.37 ± 0.11	0.30 ± 0.06
Gln	0.44 ± 0.12	0.47 ± 0.09
Gly	0.11 ± 0.05	0.26 ± 0.08
Asc	0.51 ± 0.22	0.56 ± 0.18
GSH	0.30 ± 0.13	0.59 ± 0.20
PE	0.50 ± 0.14	0.54 ± 0.11
Scy	0.08 ± 0.02	0.11 ± 0.05
tCho	0.31 ± 0.07	0.32 ± 0.06

## Discussion

The purpose of this study was to evaluate the feasibility and applicability of a novel ME-EP-JRESI sequence in human brain at 3 T. We demonstrated the implementation of the ME-EP-JRESI sequence in the brain and also the quantitation of the 2D J-resolved spectra using a modified version of ProFit algorithm. Conventional EPSI read-out facilitates inherent acceleration of one of the spatial and spectral dimensions. Using two EPI-read-out trains differently spatially encoded and separated by a refocusing 180° RF pulse, we demonstrated that a further acceleration of the 4D EP-JRESI sequence is feasible for brain applications *in*-*vivo* while maintaining sufficient spectral and spatial quality, bringing it closer to clinical application. Using multiple spin-echoes within a single TR has the same effect of filling k-space with different TEs. The same technique can be used to improve spatial resolution of 4D EP-JRESI without consuming additional scan time and producing minimal artifacts. A detail discussion on some of these aspects can be found in our earlier publications^29, 30^.

We first validated our acquisition approach using a brain phantom followed by the ProFit quantitation, demonstrating the feasibility of extending the multi echo based approach to the 4D EP-JRESI sequence. A quantitative comparison was done with the EP-JRESI phantom datasets acquired with the same parameters. As shown in Figs 3 and 4, the overall quality, localization and resolution of the ME-EP-JRESI spectra were comparable to the EP-JRESI dataset indicating a successful implementation. The spectra were localized well within the PRESS localized (white) box. The metabolite maps of the 2D peaks Cr, Cho, NAA and Glx (Fig. 3(e)) from ME-EP-JRESI and the extracted spectra exhibited similar spatial and spectral profiles as those of EP-JRESI despite the added *T*_2_-weighting during the 2^nd^ EPI-train read-outs. A little leakage can be seen in the metabolite maps from phantom data. This may be due to the chemical shift artifacts. Also, it is to be noted that no outer volume saturation bands were used when acquiring the phantom data. ProFit analysis of the phantom data showed ME-EP-JRESI and EP-JRESI gave comparable ratios close to original phantom concentrations in most of the metabolites. In addition to the main metabolites, the results indicate stable estimation of important metabolites like Glu, Gln, GABA, GSH, Asp, or PE. The CVs of most of the metabolites were under 25% with successful quantitation rate of 50% and CRLB well within 10%. The CVs are slightly high due to the fact that we averaged the values over a larger area (4 × 4 voxels) across the 10 measurements. Reproducibility of ME-EP-JRESI and EP-JRESI was high as indicated by high value of ICC. In general, the detected concentrations are slightly underestimated in ProFit which has been mentioned in the original ProFit paper by Schulte *et al*.^33^. This is especially true for some of the J-coupled metabolites. GABA and mI ratios were slightly overestimated, possibly resulting from using a TR of 2 s, a time during which metabolites are not fully recovered. In addition, it is to be noted that we have not used T_1_ or T_2_ correction. However, the ProFit results were very consistent and reproducible as evident by lower SD.

Since the outer parts of k-space in the ME-EP-JRESI sequence are acquired using the 2^nd^ EPI-train in each TR, the inner phase-encoded *k*-space lines have a shorter effective TE than the *k*-space lines on the periphery. As a result, there likely will be an intrinsic T_2_ weighting on metabolites with shorter T_2_ relaxation times, like Glx further complicated by the displacement artifacts common to chemical shift imaging sequences. However, we compared metabolites ratios from ME-EP-JRESI and EP-JRESI from a boundary voxel in each of the 10 phantom measurements and found fairly strong positive correlations. This may be because central parts of k-space, where more signal is expected, are the least affected by overall T_2_ attenuation.

The feasibility of ME-EP-JRESI implementation in human brain was further validated in a pilot study with 10 healthy controls. For quantitation of the *in*-*vivo* data, ProFit was used with an enhanced version of the basis set consisting of macromolecules and lipids apart from metabolites of interest. This was expected to give a better estimation of the respective metabolites. Both in the occipital and temporal lobes, we were able to quantify a large number of metabolite resonances reliably^5, 27^, including cross-peaks due to *J*-coupling not observable with 1D MRS. Most metabolites with higher physiological concentrations had acceptable CV’s (<25%) and CRLBs between 2–10%. In addition to the major metabolites, the results indicate stable estimation of important metabolites like Gln, Asp, PE, GSH, GABA, and Lac, which is particularly promising. This may make possible for accurate and reliable investigation of the roles of these metabolites in specific diseases in future clinical studies, including oncological studies of the brain^26, 27^. When assuming a creatine concentration of 5–9 mM, most of the metabolite values indicated in Table 2 were in good agreement with concentrations reported in the literature^37^, with the exception of Asp, NAAG and PE being overestimated. An overestimation might reflect contamination from overlapping metabolites. Due to their relatively low concentrations and overlap with other metabolites, and weak water suppression, estimation of certain metabolites like Gln, NAAG, Lac, Ala or Glc was more difficult and sometimes compromised. Moreover, the inter-subject *in*-*vivo* results demonstrate that ProFit estimation of metabolite ratios for low concentration metabolites PE, Lac, GSH, Asp or GABA were strongly dependent on the shim quality of the data, and that might be the reason for the high CVs and CRLBs in our *in*-*vivo* inter-subject measurements. Other reasons for high CV in *in*-*vivo* data compared to phantom include the additional individual differences in concentrations between the various volunteers and partial volume effects. Water line-widths of the ME-EP-JRESI data were in the range of 4 to 7 Hz in phantom and 12 to 19 Hz *in*-*vivo*. Compared to phantom studies, spectral quality was somewhat worse in the human brain due to contamination from water and extraneous lipid originating from bone marrow or subcutaneous fat close to the skull, and poor line shape.

For some of the J-coupled metabolites, there were few over- or under-estimations of metabolite ratios by the ProFit algorithm both for phantom and *in*-*vivo* data, which could be due to several factors: 1) the TR was not long enough for the magnetization of different metabolites to recover fully; 2) Differential T_2_-weighting occurred along the incremented t_1_ dimension for encoding the 2nd spectral dimension; or 3) Signal overlap, even at the 3 T field strength. In agreement with the previous work^33, 34^, few metabolites such as Glc, GSH, GABA, Ala and Lac were not reliably and successfully detected in the phantom. Overall, this study demonstrates the feasibility of accelerating the 4D EP-JRESI sequence by a factor of 2 and its applicability of human brain study *in*-*vivo*.

Though the 4D ME-EP-JRESI sequence is shown to have very similar performance compared to EP-JRESI, there are some concerns to be taken into account. As discussed by Furuyama *et al*., in spite of the well-known Fourier relationship that sampling more time points improves spectral resolution, it has been shown that sampling points after the signal has sufficiently decayed does not improve the spectral resolution. Consequently, the *in*-*vivo* EPSI readout train duration can be significantly reduced to improve the spatial resolution without sacrificing spectral resolution^29^. Further investigations will be essential to confirm this for ME-EP-JRESI and its effect on ProFit quantitation. An improved version of ProFit has been implemented recently^43^. This may adjust some of the quantitation anomalies.

It is noteworthy to mention that several other acceleration techniques have been used to reduce the scan time in MRSI. Effort had been made to acquire fewer phase encoding steps in MRSI through partial Fourier acquisition or parallel acquisition schemes, like sensitivity encoding (SENSE)^44^, but failed to accelerate the acquisition of the indirect spectral dimension. Non-uniform sampling (NUS) combined with compressed sensing (CS) reconstruction, widely used in MRI^45^, is another acceleration technique that has been attempted recently for MRSI^46–48^. CS provides greater acceleration as the undersampling can be achieved in multiple dimensions through different masking schemes. Hu *et al*. employed blipped phase encoding during the EPSI readout to nonuniformly sample the spatial as well as spectral dimensions for use in hyperpolarized 13C spectroscopic imaging^46^. We have shown earlier the potential of 2x acceleration for 4D EP-JRESI in human brain using NUS along *k*_*y*_-*t*_*1*_ dimensions^47, 48^. As scan time is still a limiting factor for the routine clinical applications of ME-EP-JRESI sequence, additional acceleration may be achieved through CS by imposing NUS along the *t*_*1*_ direction. This may add additional artifacts, but future work should be pursued along this direction to study the feasibility of this method. Another future direction to look at is to extend the 4D ME-EP-JRESI sequence to 5D (3 spatial + 2 spectral), by adding another spatial dimension (*z*), which will enable covering nearly the full brain in a single record. This can be achieved by the utilization of NUS along the (*k*_*z*_, *t*_*1*_) plane and subsequent CS reconstruction as implemented by Wilson *et al*.^49^ for EP-JRESI.

## Conclusions

We have shown that the use of twice phase encoded EPI-readout within a single TR can reduce the scan time of the EP-JRESI sequence. Though we used only two echoes, more EPI-readout trains can in theory be used to improve spatial resolution even though there will be increased T_2_* losses at higher frequency k-space points^29^. The present ME-EP-JRESI sequence has been tested on human brain in a clinical setting and shown good spectral sensitivity and spatial specificity. Reproducibility studies using the physiological phantom data demonstrate reliability comparable to existing techniques like EP-JRESI. Further optimization of the ME-EP-JRESI sequence using parallel imaging or compressed sensing may allow even greater reduction in scan times or higher imaging resolution though there may be some compromise on overall spectral quality.
